# Pica and refractory iron deficiency anaemia: a case report

**DOI:** 10.1186/1752-1947-2-324

**Published:** 2008-10-06

**Authors:** Christophe von Garnier, Holger Stünitz, Michael Decker, Edouard Battegay, Andreas Zeller

**Affiliations:** 1 Medical Outpatient Department, University Hospital Basel, Petersgraben, 4031 Basel, Switzerland; 2 Respiratory Medicine, University Hospital Bern, 3010 Bern, Switzerland; 3 Department of Geosciences, University of Basel, Bernouillistrasse, 4056 Basel, Switzerland; 4 Division of Haematology, University Hospital of Basel, Petersgraben, 4031 Basel, Switzerland

## Abstract

**Introduction:**

Iron deficiency is the most common cause of anaemia worldwide. Pica, the ingestion of substances that are inappropriate for consumption, is associated with iron deficiency and may be under-diagnosed.

**Case presentation:**

A 34-year-old woman presented with iron deficiency anaemia refractory to treatment for more than a decade. The clinical presentation, endoscopic findings and laboratory investigations were consistent with pica. Subsequent geophysical analysis confirmed that the ingested material was kaolin, a negatively charged silicate.

**Conclusion:**

Prolonged unexplained iron deficiency anaemia should prompt clinicians to remember and inquire about pica. In our patient, this would have averted numerous unnecessary investigations and prevented a decade-long suffering.

## Introduction

Pica refers to an ill-defined entity known as a perverted appetite for substances inappropriate for consumption, such as kaolin (geophagia) [1]. Cultural factors may influence this dietary behaviour, which can even lead to serious complications that require surgical intervention [2,3]. Kaolinite is the most important component of kaolin, a white chalky silicate used in the paper-coating industry, in ceramics production and in the pharmaceutical industry. The negatively charged surface of kaolinite is able to exchange and adsorb cations (for example, Fe^2+ ^and Fe^3+^) in the duodenum, where iron absorption occurs. As a consequence, iron deficiency anaemia may be associated with the ingestion of kaolin. We report the case of an African woman who had lived in Europe for more than a decade and presented with a 12-year history of seemingly refractory iron deficiency anaemia.

## Case presentation

A 22-year-old African woman first presented to our medical outpatients department in 1993 with anaemia, with test results as follows: haemoglobin (Hb) 96 g/litre (normal range 120 to 160 g/litre), mean corpuscular volume (MCV) 74.7 fl (normal range 79 to 95 fl), mean corpuscular haemoglobin concentration (MCHC) 313 g/litre (normal range 320 to 360 g/litre), reticulocytes 13‰ (normal range 5 to15‰) and ferritin of 9 ng/ml (normal range 10 to 200 ng/ml). Iron deficiency was the most likely cause, but further investigations (occult stool blood test, gastroscopy, colonoscopy, gynaecological examination, Hb electrophoresis) failed to show an obvious bleeding source or abnormal Hb. Despite oral iron replacement therapy (160 mg of Fe^2+ ^daily), Hb values remained low (100 to 115 g/litre) over the next 4 years, after which the patient was lost to follow-up.

Twelve years later, in 2005, the patient presented with incapacitating fatigue related to severe iron deficiency anaemia (Hb 75 g/litre, MCV 64.6 fl, MCHC 299 g/litre, reticulocytes 13‰, ferritin 6 ng/ml and soluble transferrin receptor 13.4 mg/ml (normal range 2.2 to 4.5 mg/ml)). Gastroscopy showed normal gastric mucosa and histological evidence of a highly active gastritis with *Helicobacter pylori *infestation in biopsy samples. A capsule endoscopy revealed several non-specific erythematous mucosal changes in the bulbus duodeni not seen during gastroscopy.

**Figure 1:**
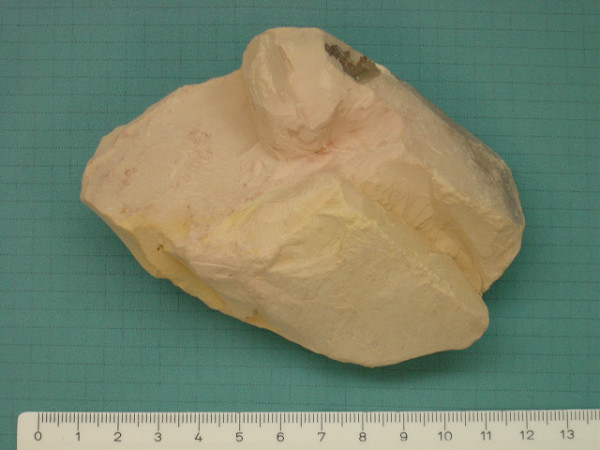
**The stone consumed by the patient**. Scale in centimetres.

**Figure 2:**
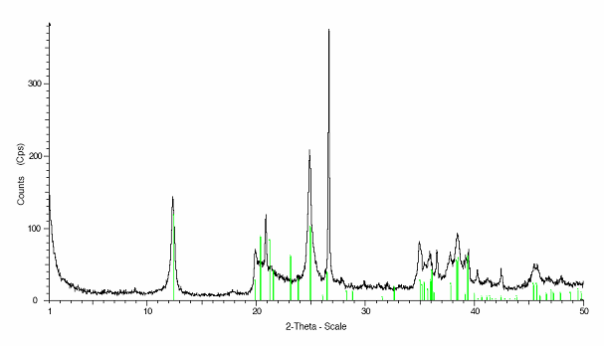
**X-ray diffraction data for the kaolin stone sample**. X-ray diffraction data of the sample material provided by the patient (Cu-Kα1 irradiation, 2θ range 1° to 50°). The vertical lines mark the kaolinite peaks, all others correspond to quartz-specific peaks.

During a follow-up visit and further explicit questioning about particular eating habits, the patient reluctantly disclosed an almost daily consumption of 'a friable stone' over more than a decade. She reported having developed a particularly strong craving for such stones, of which she would suck on small pieces until these completely dissolved. She had acquired this habit 15 years ago in her home country Cameroon, where consumption of stones is common. To characterise the chalky stone (Figure 1), we contacted our geosciences department. X-ray diffraction measurements confirmed that the substance was essentially composed of kaolinite with traces of quartz (Figure 2). After cessation of kaolinite ingestion, we administered intravenous iron replacement therapy (total of 1000 mg) and the anaemia was corrected within 1 month (Hb 125 g/litre, MCV 79.4 fl, MCHC 333 g/litre, ferritin 13 ng/ml, soluble transferrin receptor 6.6 mg/ml). Tests 3 months later showed a stable blood count and iron studies as follows: Hb 120 g/litre, MCV 83.6 fl, MCHC 346 g/litre, ferritin 11 ng/ml and soluble transferrin receptor 6.0 mg/ml.

## Discussion

The word pica comes from the Latin word for magpie, a bird known for its unusual eating habits. Pica is characterised by persistent craving and compulsive eating of non-food substances. Pica in humans has many different subgroups, defined by the substance that is ingested. Examples of pica are eating earth, soil or clay (geophagia) or ice (pagophagia). The prevalence and type of pica depend on ethnic and cultural factors that affect dietary practices [4]. Pica has been reported to be associated with severe iron deficiency anaemia in up to half of patients; however, it is unclear whether pica causes or is the consequence of iron deficiency anaemia [5-8]. In our case, there are several reasons why kaolin ingestion may have caused iron deficiency anaemia. First, experimental data from kaolin-fed pregnant rats showed significant maternal anaemia and reduced birth weight, both of which were prevented in the iron-supplementation control group [9]. Second, adsorption of Fe^2+ ^and Fe^3+ ^to the negatively charged and large active surface area of kaolinite may lead to a reduction of available iron in the duodenum. As a consequence, the absorption of iron might decrease, resulting in iron deficiency. Third, traces of quartz commonly found in kaolinite may cause abrasion and favour increased mucosal sloughing and iron loss. Fourth, kaolin consumption and geophagia may cause parasitic infestation and further iron loss [7-10].

Although pica is a rare condition in central Europe [11], it may be underestimated in multicultural societies and it is therefore important to remember and inquire about particular eating habits in the context of unexplained iron deficiency anaemia. Thus, proper history remains the most inexpensive investigation.

## Conclusion

In the context of an unexplained iron deficiency anaemia, it is important to remember and inquire about pica. In our patient, this would have averted numerous unnecessary investigations and prevented a decade of suffering.

## Abbreviations

Hb: haemoglobin; MCHC: mean corpuscular haemoglobin concentration; MCV: mean corpuscular volume.

## Competing interests

The authors declare that they have no competing interests.

## Consent

Written informed consent was obtained from the patient for publication of this case report and any accompanying images. A copy of the written consent is available for review by the Editor-in-Chief of this journal.

## Authors' contributions

CVG managed the patient, conceived the initial idea and drafted the paper. HS performed and interpreted the geophysical investigations. MD reviewed and interpreted the full blood counts and iron studies. EB critically reviewed and interpreted the investigations. AZ managed the patient and critically reviewed the manuscript. All authors read and approved the final manuscript.
